# Association Between Sleep-Related Leg Movements and Diabetes Prevalence: A Cohort Study From the United States

**DOI:** 10.7759/cureus.84701

**Published:** 2025-05-23

**Authors:** Qinglan Ding, Yuting Xie, Brian B Koo, Zachary Hass, Brian Wojeck, Jason J Sico, Laura E Murray-Kolb, Dawn M Bravata, Andrey Zinchuk

**Affiliations:** 1 School of Nursing, College of Health and Human Sciences, Purdue University, West Lafayette, USA; 2 National Collaborating Center for Neurological Disorders Prevention, Beijing Tiantan Hospital, Capital Medical University, Beijing, CHN; 3 Neurology, Yale School of Medicine, New Haven, USA; 4 School of Industrial Engineering, Purdue University, West Lafayette, USA; 5 Endocrinology, Yale School of Medicine, New Haven, USA; 6 Nutrition Science, College of Health and Human Sciences, Purdue University, West Lafayette, USA; 7 Internal Medicine and Neurology, Indiana University School of Medicine, Indianapolis, USA; 8 Pulmonary, Critical Care, and Sleep Medicine, Yale School of Medicine, New Haven, USA

**Keywords:** diabetes mellitus, mediation analysis, periodic limb movements, racial differences, sleep-related leg movements

## Abstract

Background

Sleep is integral to cardiometabolic health. While there is emerging evidence linking sleep-related leg movements (SRLM) to diabetes mellitus (DM), the underlying mechanisms remain unclear. This study investigates the association between SRLM and DM prevalence in a national population-based cohort study in the United States (US), considering potential mediators like short sleep duration and inflammation and examining variations across age, sex, and race/ethnicity.

Methods

We analyzed data from 9,191 adults (aged ≥18 years) from the National Health and Nutrition Examination Survey (NHANES) 2005-2008. We assessed the frequency of SRLM (leg jerks and leg cramps) and diabetes prevalence (self-reported diagnosis, diabetes medication use, or glycosylated hemoglobin (HbA1c) ≥ 6.5%). Weighted logistic regression models were used to evaluate the associations, adjusting for demographic and clinical-related confounders. Mediation analyses were conducted to explore the roles of short sleep duration and inflammation (C-reactive protein levels).

Results

The study revealed that 1,278 (14%) participants have DM. SRLM was associated with a 72% increase in the odds of diabetes (OR=1.72, 95% CI: 1.06-2.81). The association is more pronounced in non-Hispanic White individuals, females, and adults aged 18-65. Short sleep duration and inflammation mediated 5.0% and 3.9% of this association, respectively.

Conclusions

SRLM is independently and linearly associated with increased prevalence of diabetes in a representative sample of the US adult population. Short sleep duration and inflammation mediated a small part of this association. Confirming the association in other samples and further investigation into its mechanisms are warranted to better understand the roles of the SRLM in the risk of diabetes.

## Introduction

Sleep is essential for maintaining cardiometabolic health [1], recognized by the American Heart Association as a modifiable factor for cardiovascular health [2]. Sleep-related leg movements (SRLM), ranging from involuntary leg twitches to pronounced limb jerks, have been identified as major disruptions to restful sleep [3]. This spectrum includes phenomena such as periodic limb movements of sleep (PLMS), marked by repetitive limb contractions during sleep, and restless legs syndrome (RLS), characterized by uncomforting leg sensations prompting an irresistible urge to move [4]. Despite their high prevalence among the elderly, the implications of SRLM for health outcomes remain relatively underexplored in the general population.

Emerging evidence suggests that SRLMs, particularly RLS, exhibit a significant association with metabolic health [5]. Research demonstrates that individuals with RLS have a significantly higher risk of developing diabetes mellitus (DM) compared to the general population [3,5-7]. Our prior study within a high-comorbidity United States (US) Veteran population shows that those exhibiting frequent PLMS are twice as likely to develop DM [8]. Despite these findings, most studies have focused primarily on RLS, potentially neglecting the broader spectrum of SRLM and how they differently affect metabolic health [9].

Additionally, the risk of DM and associated metabolic dysfunction is often influenced by an individual’s age, sex, and race/ethnicity [10]. However, the degree to which these demographic factors influence the relationship between SRLM and metabolic well-being remains poorly understood. Addressing this research gap is important for a detailed understanding of health disparities and ensuring equitable health outcomes across diverse populations.

While the associations between RLS, a type of SRLM, and DM are well-documented, the underlying mechanisms, particularly the role of risk factors such as short sleep duration and inflammation, are not thoroughly understood [11]. SRLM may intensify systemic inflammation [11], disrupting normal sleep patterns and contributing to sleep disturbances such as insomnia or SRLM-induced arousals. In turn, systemic inflammation is a recognized driver of type 2 DM pathogenesis, affecting critical risk factors such as obesity, hyperglycemia, and hypertension, and triggering pro-inflammatory cytokine pathways in insulin-sensitive tissues [12]. These observations imply that inflammation could mediate the relationship between SRLM and DM, indicating a complex interplay that warrants further examination [12].

In response to these research gaps, we performed a cross-sectional analysis to investigate the association between SRLM and the prevalence of DM. We also explored potential underlying mechanisms, such as sleep duration and inflammation, that could mediate the association between SRLM and DM. Additionally, we explored variations in the associations between SRLM and DM prevalence among different age groups, sexes, and various racial/ethnic groups.

## Materials and methods

Data sources and study population

**Figure 1:**
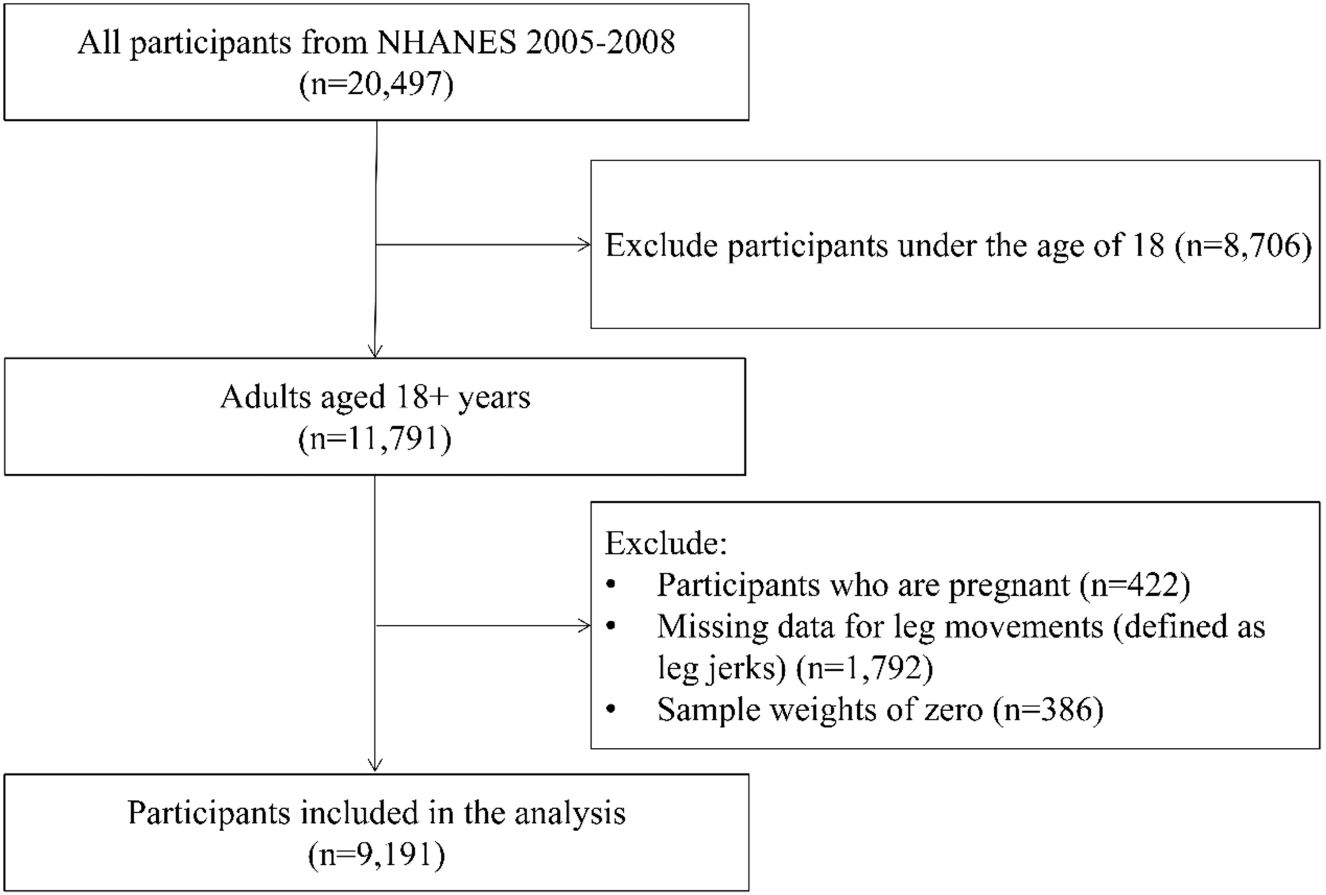
Selection of participants in primary analysis NHANES: National Health and Nutrition Examination Survey

This analysis utilized data from the National Health and Nutrition Examination Survey (NHANES) 2005-2008 cycle due to the availability of sleep variables. NHANES is a nationally representative cross-sectional survey conducted by the Centers for Disease Control and Prevention [13]. Participants were selected using a stratified, multistage probability cluster sampling design. The details of NHANES have been published previously [13]. Ethical approval for the NHANES protocol was obtained from the National Center for Health Statistics Research Ethics Review Board, and written informed consent was obtained from participants. Because all data analyzed by investigators were de-identified and publicly available, the Purdue University Committee on Human Research determined that further IRB review was not required. For this analysis, we excluded pregnant individuals, those under 18 years of age, missing data for leg movement, and sample weights of zero (Figure 1).

Definition of sleep-related leg movements

During the NHANES interview, participants were asked about the frequency of two types of SRLM (leg jerks and leg cramps) experienced during sleep in the past month. Response options ranged from “never” to “almost always”, with corresponding frequency categories provided. We defined SRLM as individuals who reported experiencing leg jerks “often” (5-15 times a month) or “almost always” (16-30 times a month) in the past month. The decision to use this definition of leg jerks as a proxy for SRLM was based on the likelihood that this question would capture a large portion of persons with either PLMS or RLS. For sensitivity analysis, we used a more expansive definition for SRLM to include individuals who reported experiencing leg jerks or leg cramps “often” or “almost always” while sleeping.

Diagnosis of diabetes mellitus

Participants were considered to have DM if they met any of the following conditions: (1) self-reporting a prior diagnosis of DM by a healthcare professional, (2) using anti-diabetic medications, or (3) had a measured glycosylated hemoglobin (HbA1c) level of 6.5% or higher [14].

Selection of potential mediators

For our exploratory mediation analyses, we included short sleep duration and systemic inflammation as mediators. Our choice of potential mediators was informed by the previous epidemiological studies on the association between SRLM, short sleep, and DM, and literature on the pathophysiological connections between SRLM, inflammation, and DM [11,12,15,16]. Short sleep duration was defined as less than seven hours per night on weekdays or workdays [17]. Systemic inflammation was quantified by C-reactive protein (CRP, mg/L) levels using the latex-enhanced nephelometry method.

Statistical analysis

Descriptive Analysis

All analyses were performed while considering the intricate NHANES sampling design and data weighting, ensuring our results represent the national population accurately. We followed NHANES guidelines and utilized the four-year combined mobile examination center weight for the 2005-2008 period, which was calculated as half of the two-year weight. We compared demographic, socioeconomic, and clinical characteristics between participants with and without DM. Continuous variables are presented as weighted mean ± standard deviation (SD), while categorical variables are expressed as unweighted frequencies and weighted percentages. Group differences were assessed using the chi-squared test for categorical variables and independent t-tests for continuous variables.

Primary Association Analyses

In our primary analyses, we utilized weighted unconditional logistic regression models to estimate odds ratios (ORs) and their corresponding 95% confidence intervals (CIs) for the association between SRLM and the prevalence of DM. In our primary analyses, covariates included age, gender, race/ethnicity (categorized as non-Hispanic White individuals, non-Hispanic Black individuals, Hispanic individuals, or other individuals), education (categorized as less than high school, high school, or more than high school), marital status (categorized as married or living with a partner, widowed, divorced, separated, or never married), health insurance (yes or no), the ratio of family income to poverty level (PIR), body mass index (BMI), history of hypertension, and sleep apnea. The PIR was calculated by dividing family income by the federal poverty level and categorized as less than 1.30, 1.30-3.49, or ≥3.50. BMI was calculated as weight in kilograms divided by height in meters squared and categorized as <18.5 kg/m^2^, 18.5-25 kg/m^2^, 25-30 kg/m^2^, or ≥30 kg/m^2^. The percentage of missing values of covariates was below 10%, and missing covariate data were categorized separately to ensure the inclusion of incomplete cases (Table 1).

**Table 1:** Frequency and percentage of missing values of covariates in primary analysis

Covariates	Variable coding	Frequency of missing (proportion of the study population)
Age	Continuous variable	0
Gender	0=male, 1=female	0
Race/ethnicity	0=Non-Hispanic White, 1=Hispanic, 2=Non-Hispanic Black, 3=Other race	0
Education	0=less than high school, 1=high school, 2=more than high school, 99=missing group	12 (0.13%)
Marital status	0=married/living with a partner, 1=widowed, 2=divorced, 3=separated, 4=never married	248 (2.70%)
Health insurance	0=no, 1=yes, 99=missing group	18 (0.20%)
Ratio of family income to poverty	0≤1.30, 1=1.30-3.49, 2≥3.50, 99=missing group	682 (7.42%)
Body mass index	0=18.5-25 kg/m^2^, 1≤18.5 kg/m^2^, 2=25-30 kg/m^2^, 3≥30 kg/m^2^, 99=missing group	178 (1.94%)
Hypertension	0=no, 1=yes, 99=missing group	1 (0.01%)
Sleep apnea	0=no, 1=yes, 99=missing group	272 (2.96%)

To further explore potential dose-response relationships between SRLM (both leg jerks only and leg jerks or cramps) and DM risk or HbA1c levels, we incorporated restricted cubic splines into regression models using the R-package rms, utilizing four knots positioned at the 5th, 35th, 65th, and 95th percentiles. We conducted tests for non-linearity using likelihood ratio tests, comparing models with only the linear term to those with both linear and cubic spline terms, with corresponding p-values indicating non-linearity.

Effect Modification Analyses

Subgroup analyses were conducted to assess whether the association between SRLM and DM varied by age (18-65 versus ≥65 years), sex, or race/ethnicity. Additionally, we explored effect modification by adding interaction terms into fully adjusted models.

Exploratory Mediation Analyses

The R package mediation was used for the exploratory mediation analyses. We first evaluated whether the SRLMs were associated with two potential mediators: short sleep duration (<7 hours/night) and inflammation (CRP levels). Subsequently, we assessed the relationship between each potential mediator and the prevalence of DM.

Sensitivity Analyses

In sensitivity analyses, we considered an alternative definition of SRLM by incorporating leg jerks or leg cramps during sleep. Additionally, we introduced iron deficiency, defined as serum ferritin levels below 75 μg/L, as an additional confounding factor in logistic regression models examining the association of SRLM with DM [18]. This analysis focused on a restricted cohort of female participants aged 18-49 years, for whom iron deficiency status was available.

All statistical analyses were completed using R (version 4.3.1, R Foundation for Statistical Computing, Vienna, Austria). A two-sided p-value < 0.05 was considered statistically significant.

## Results

Characteristics of participants

Table 2 provides a comprehensive overview of the demographics and clinical characteristics of our study participants. The mean age (SD) of the overall sample was 46.10 (19.58) years, with 4,528 (51.08%) participants being women. Among the total cohort, 2,423 (12.50%) identified as Hispanic individuals, 4,216 (69.83%) as non-Hispanic White individuals, and 2,194 (12.18%) as non-Hispanic Black individuals. A total of 1,278 (13.90%) had prevalent DM. Participants with DM tended to be older and were more likely to identify as non-White individuals. They also had lower educational attainment (high school or lower), were more frequently widowed, divorced, or separated, had a higher frequency of health insurance coverage, and had a lower PIR, indicating their income was less than the federal poverty level. Additionally, individuals with DM exhibited a higher BMI, a history of hypertension, a greater prevalence of sleep apnea, shorter sleep duration, and elevated levels of CRP.

**Table 2:** Characteristics of study participants with and without DM in the NHANES 2005-2008 All percentages and p-values were calculated by excluding missing data. ^†^ T-statistic value for continuous variables, chi-square value for categorical variables. ^* ^p-values were calculated to compare the differences between participants with and without DM. DM: diabetes mellitus; PIR: family income to poverty ratio; CRP: C-reactive protein; NHANES: National Health and Nutrition Examination Survey

Characteristics	Total (n=9,191)	DM		Statistic value^†^	p-value^*^
		Yes (n=1,278)	No (n=7,913)		
Age (years), mean±SD	46.10±19.58	59.03±13.80	44.66±19.47	27.16	<0.001
Age group (years), n (%)				151.29	<0.001
18-65	6,966 (82.980)	698 (61.94)	6,268 (85.32)		
≥65	2,225 (17.02)	580 (38.06)	1,645 (14.68)		
Gender, n (%)				0.02	0.883
Male	4,663 (48.92)	651 (49.20)	4,012 (48.89)		
Female	4,528 (51.08)	627 (50.80)	3,901 (51.11)		
Race/ethnicity, n (%)				47.63	<0.001
Hispanic	2,423 (12.50)	343 (13.48)	2,080 (12.39)		
Non-Hispanic White	4,216 (69.83)	478 (60.63)	3,738 (70.85)		
Non-Hispanic Black	2,194 (12.18)	412 (19.64)	1,782 (11.35)		
Other race	358 (5.49)	45 (6.24)	313 (5.40)		
Education, n (%)				58.31	<0.001
Less than high school	2,800 (19.78)	528 (28.39)	2,272 (18.82)		
High school	2,300 (25.69)	320 (27.46)	1,980 (25.38)		
More than high school	4,079 (54.53)	428 (43.15)	3,651 (55.80)		
Marital status, n (%)				93.56	<0.001
Married/living with a partner	5,149 (63.42)	754 (63.25)	4,395 (63.44)		
Widowed	812 (6.32)	215 (14.02)	597 (5.45)		
Divorced	901 (10.13)	154 (12.13)	747 (9.90)		
Separated	279 (2.36)	46 (2.42)	233 (2.36)		
Never married	1,802 (17.76)	105 (8.18)	1,697 (18.85)		
Health insurance, n (%)	7,022 (80.83)	1,086 (87.40)	5,936 (80.10)	26.06	<0.001
PIR, n (%)				24.17	<0.001
<1.30	2,533 (19.55)	384 (23.91)	2,149 (19.08)		
1.30-3.49	3,307 (36.31)	491 (41.42)	2,816 (35.76)		
≥3.50	2,669 (43.14)	273 (34.67)	2,396 (45.16)		
Body mass index, n (%)				170.44	<0.001
<18.5 kg/m^2^	178 (1.86)	4 (0.34)	174 (2.02)		
18.5-25 kg/m^2^	2,660 (31.23)	167 (12.69)	2,493 (33.26)		
25-30 kg/m^2^	3,018 (33.30)	350 (26.84)	2,668 (34.01)		
≥30 kg/m^2^	3,157 (33.61)	707 (60.13)	2,450 (30.71)		
Hypertension, n (%)	4,573 (47.02)	1,007 (76.76)	3,566 (43.72)	157.76	<0.001
Sleep apnea, n (%)	373 (4.42)	126 (12.77)	247 (3.50)	26.35	<0.001
Short sleep duration, n (%)	3,480 (36.03)	527 (42.05)	2,953 (35.36)	15.58	<0.001
CRP (mg/dL), mean±SD	0.39±0.80	0.62±1.01	0.37±0.75	5.34	<0.001
Iron deficiency, n (%)	1,797 (78.34)	69 (64.78)	1,728 (78.92)	4.35	0.045

Associations between sleep-related leg movements and diabetes mellitus prevalence

**Figure 2:**
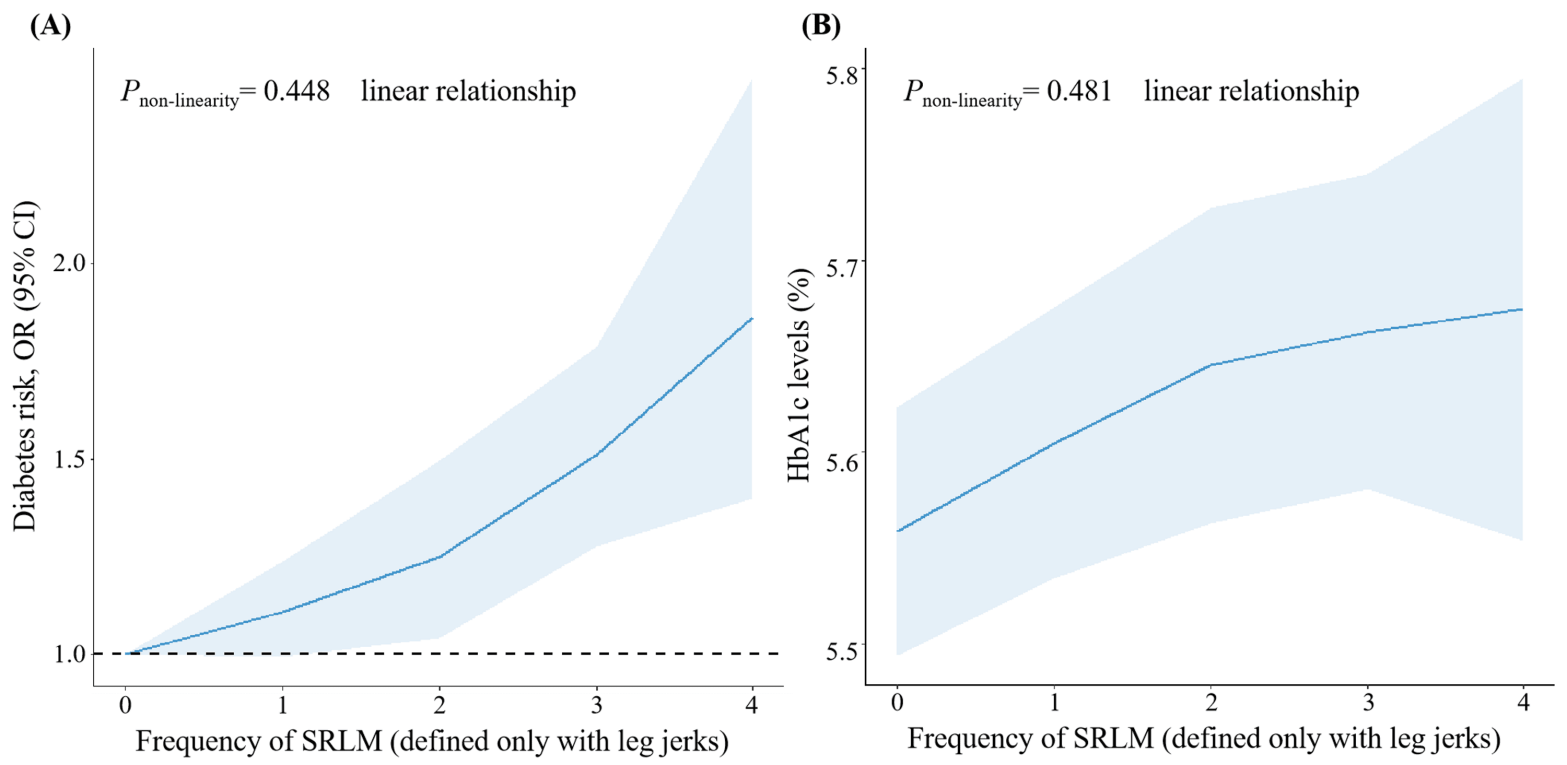
Dose-response relationship between SRLM (defined only with leg jerks) and risk of DM or HbA1c levels based on data from NHANES Multivariate logistic regression models were adjusted for age, gender, race/ethnicity, education, marital status, insurance, family income to poverty ratio, body mass index, hypertension, and sleep apnea. SRLM (defined only with leg jerks) frequency: 0 represents never, 1 represents rarely (1 time/month), 2 represents sometimes (2-4 times/month), 3 represents often (5-15 times/month), and 4 represents almost always (16-30 times/month). SRLM: sleep-related leg movements; DM: diabetes mellitus; OR: odds ratio; CI: confidence interval; HbA1c: glycosylated hemoglobin; NHANES: National Health and Nutrition Examination Survey

In the entire sample, participants with SRLM exhibited higher odds of a DM diagnosis compared to those without SRLM in the unadjusted model, with an OR of 1.88 (95% CI: 1.36-2.59) (Table 3). A statistically significant association between SRLM and prevalent DM persisted (OR 1.72, 95% CI: 1.06-2.81) in a fully adjusted model (Table 3, Model 2). Notably, a significant linear trend was evident, signifying consistent associations between SRLM and both DM prevalence and HbA1c levels (p-value for non-linearity is 0.448 for DM prevalence and p-value for non-linearity is 0.481 for HbA1c). This linear trend suggests that the risk of DM and HbA1c levels increased with a higher frequency of SRLM (Figure 2).

**Table 3:** Associations between SRLM (defined only with leg jerks) and risk of DM Model 1: Adjusted for socio-demographics, including age, gender, race/ethnicity, education, marital status, insurance, and family income to poverty ratio. Race, gender, and age were removed from the adjusted covariates of the corresponding stratified analyses. Model 2: Further adjusted for body mass index, hypertension, and sleep apnea based on Model 1. The analysis models of the total population and subgroups of the population were both based on the reference group without SRLM. ^†^ p-values for interaction were calculated based on Model 2. * p<0.05 SRLM: sleep-related leg movements; DM: diabetes mellitus; OR: odds ratio; CI: confidence interval

Group	DM		Unadjusted model: OR (95% CI)	Model 1: OR (95% CI)	Model 2: OR (95% CI)	p-value for interaction^†^
	Yes	No				
SRLM (ref=no)						
Total population	1,278	7,913	1.88 (1.36, 2.59)*	1.98 (1.34, 2.91)*	1.72 (1.06, 2.81)*	
Subgroup						
Stratified by race/ethnicity						0.544
(Ref=no SRLM)						
Hispanic	343	2,080	2.25 (1.17, 4.33)*	1.87 (0.84, 4.16)	1.28 (0.57, 2.91)	
Non-Hispanic Whites	478	3,738	2.08 (1.45, 2.97)*	2.16 (1.43, 3.25)*	2.04 (1.31, 3.20)*	
Non-Hispanic Blacks	412	1,782	1.53 (0.86, 2.74)	1.39 (0.70, 2.76)	1.38 (0.65, 2.93)	
Other race	45	313	1.40 (0.35, 5.68)	1.07 (0.19, 6.12)	0.68 (0.10, 4.63)	
Stratified by gender						0.646
(Ref=no SRLM)						
Male	651	4,012	1.63 (1.00, 2.67)	1.71 (0.91, 3.19)	1.61 (0.74, 3.50)	
Female	627	3,901	2.09 (1.50, 2.92)*	2.23 (1.49, 3.32)*	1.80 (1.17, 2.75)*	
Stratified by age						0.103
(Ref=no SRLM)						
18-65 years	698	6,268	2.46 (1.60, 3.76)*	2.52 (1.58, 4.01)*	2.09 (1.17, 3.75)*	
≥65 years	580	1,645	1.05 (0.68, 1.63)	1.06 (0.66, 1.67)	0.97 (0.58, 1.62)	

Subgroup analyses, stratified by race/ethnicity, sex, and age, revealed that the association between SRLM and DM was pronounced in non-Hispanic White participants, female participants, and individuals aged between 18 and 65 years (p<0.05) (Table 3).

Exploratory mediation analysis for the association between sleep-related leg movements and diabetes mellitus

**Figure 3:**
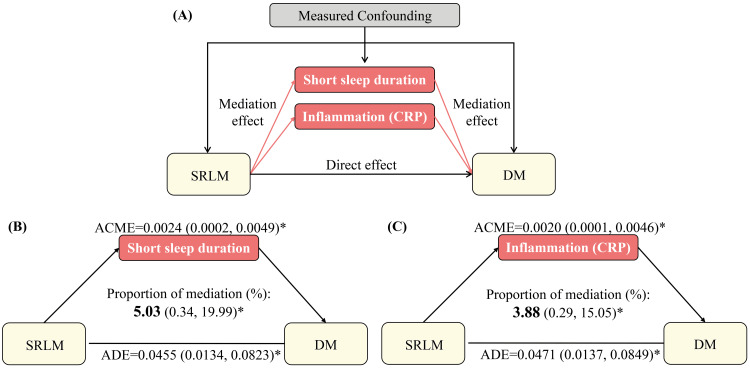
Path diagram of the mediation analysis of the association between SRLM and DM among total population Analyses in the total population were adjusted for sociodemographics (age, gender, race/ethnicity, education, marital status, insurance, and family income to poverty ratio), body mass index, hypertension, and sleep apnea. The graph in (A) represented the schematic of the mediation analysis; those in (B) and (C) depicted the mediating effects of short sleep duration and inflammation, respectively. * p<0.05 SRLM: sleep-related leg movements; DM: diabetes mellitus; ADE: average direct effect; ACME: average causal mediation effect

Mediation analysis by short sleep duration showed a small but statistically significant mediating role of short sleep duration in the association between SRLM and the prevalence of DM. After adjusting for sociodemographics and clinical characteristics, short sleep duration mediated 5.0% of the relationship between SRLM and DM (Figure 3). Notably, short sleep duration mediated this association for non-Hispanic White participants and females only (Table 4). Among non-Hispanic White participants, short sleep duration mediated 9.8% of the association between SRLM and the prevalence of DM. Additionally, in female participants, it mediated 6.4% of the association (Table 4).

**Table 4:** Mediation analysis for the association between SRLM (defined only with leg jerks) and risk of DM among race/ethnicity, gender and age subgroups Analyses of the total population were adjusted for sociodemographics (age, gender, race/ethnicity, education, marital status, insurance, and family income to poverty ratio), body mass index, hypertension, and sleep apnea. Race, gender, and age were removed from the adjusted covariates of the corresponding stratified analyses. ^†^ Nonsignificant proportions of mediation were not shown. * p<0.05 SRLM: sleep-related leg movements; DM: diabetes mellitus; CRP: C-reactive protein; ADE: average direct effect; ACME: average causal mediation effect; CI: confidence interval

Mediator	DM		Total effect		ADE		ACME		Proportion mediated (%)^†^
	Yes	No	β	95% CI	β	95% CI	β	95% CI	
Short sleep duration									
Hispanic	343	2,080	0.0222	-0.0340, 0.0931	0.0274	-0.0299, 0.1026	-0.0052	-0.0126, -0.0004	-
Non-Hispanic Whites	478	3,738	0.0587	0.0261, 0.0950	0.0529	0.0197, 0.0899	0.0057	0.0020, 0.0899	9.79 (2.94, 25.89)*
Non-Hispanic Blacks	412	1,782	0.0378	-0.0250, 0.1102	0.0382	-0.0258, 0.1116	-0.0004	-0.0039, 0.0024	-
Other race	45	313	-0.0205	-0.1126, 0.1270	-0.0180	-0.1117, 0.1290	-0.0025	-0.0150, 0.0053	-
Male	651	4,012	0.0448	-0.0100, 0.1125	0.0433	-0.0123, 0.1109	0.0015	-0.0031, 0.0071	-
Female	627	3,901	0.0505	0.0217, 0.0824	0.0472	0.0179, 0.0798	0.0033	0.0006, 0.0066	6.42 (0.94, 22.45)*
18-65 years	698	6,268	0.0578	0.0191, 0.1017	0.0555	0.0164, 0.0985	0.0022	-0.0005, 0.0055	-
≥65 years	580	1,645	-0.0045	-0.0631, 0.0570	-0.0052	-0.0628, 0.0570	0.0007	-0.0035, 0.0061	-
Inflammation (CRP)									
Hispanic	330	1,965	0.0246	-0.0325, 0.0971	0.0239	-0.0321, 0.0947	0.0008	-0.0027, 0.0052	-
Non-Hispanic Whites	460	3,547	0.0607	0.0253, 0.0995	0.0584	0.0235, 0.0972	0.0023	0.0001, 0.0057	3.55 (0.05, 11.55)*
Non-Hispanic Blacks	374	1,592	0.0344	-0.0328, 0.1161	0.0369	-0.0292, 0.1186	-0.0025	-0.0069, 0.0010	-
Other race	43	294	-0.0197	-0.1161, 0.1256	-0.0202	-0.1201, 0.1313	0.0004	-0.0091, 0.0104	-
Male	625	3,752	0.0399	-0.0208, 0.1132	0.0359	-0.0243, 0.1083	0.0040	0.0004, 0.0096	-
Female	582	3,646	0.0570	0.0248, 0.0904	0.0566	0.0245, 0.0901	0.0004	-0.0013, 0.0024	-
18-65 years	668	5,851	0.0598	0.0189, 0.1054	0.0572	0.0156, 0.1019	0.0026	-0.0002, 0.0067	-
≥65 years	539	1,547	-0.0027	-0.0618, 0.0620	-0.0005	-0.0604, 0.0651	-0.0022	-0.0085, 0.0025	-

We also examined the potential mediating role of inflammation, as measured by CRP, in a subset of 8,605 individuals after excluding those with missing CRP values (n=586). Upon adjusting for sociodemographic and clinical characteristics, our analyses showed that CRP mediated 3.9% of the association between SRLM and the prevalence of DM (Figure 3). Significant race/ethnicity variations were observed, with CRP mediating 3.55% of the association between SRLM and the prevalence of DM among non-Hispanic White participants (Table 4).

Sensitivity analysis

**Figure 4:**
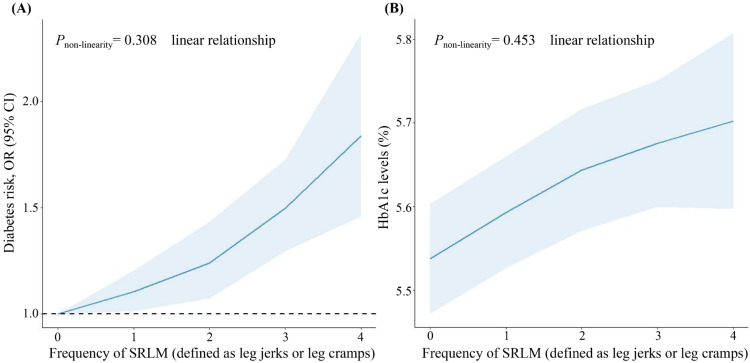
Dose-response relationship between SRLM (defined as leg jerks or leg cramps) and risk of DM or HbA1c levels based on data from NHANES Multivariate logistic regression models were adjusted for age, gender, race/ethnicity, education, marital status, insurance, family income to poverty ratio, body mass index, hypertension, and sleep apnea. SRLM (defined as leg jerks or leg cramps) frequency: 0 represents never, 1 represents rarely (1 time/month), 2 represents sometimes (2-4 times/month), 3 represents often (5-15 times/month), and 4 represents almost always (16-30 times/month). SRLM: sleep-related leg movements; DM: diabetes mellitus; OR: odds ratio; CI: confidence interval; HbA1c: glycosylated hemoglobin; NHANES: National Health and Nutrition Examination Survey

When defining SRLM as leg jerks or leg cramps during sleep, a total of 7,570 adults meeting the inclusion criteria (non-zero sample weights, non-pregnant status, and available SRLM responses) were included in the sensitivity analysis, and similar associations between SRLM and risk of DM incidence were noted (Table 5, Figure 4). We also observed mediation by short sleep duration and inflammation in this association (Table 6).

**Table 5:** Sensitivity analysis of the association between SRLM (defined as leg jerks or leg cramps) and risk of DM Model 1: Adjusted for socio-demographics, including age, gender, race/ethnicity, education, marital status, insurance, and family income to poverty ratio. Model 2: Further adjusted for body mass index, hypertension, and sleep apnea based on Model 1. Race, gender, and age were removed from the adjusted covariates of the corresponding stratified analyses. ^†^ p-values for interaction were calculated based on Model 2. * p<0.05 SRLM: sleep-related leg movements; DM: diabetes mellitus; OR: odds ratio; CI: confidence interval

Group	DM		Unadjusted model: OR (95% CI)	Model 1: OR (95% CI)	Model 2: OR (95% CI)	p-value for interaction^†^
	Yes	No				
SRLM (ref=no)						
Total population	985	6,585	2.34 (1.77, 3.11)*	1.99 (1.45, 2.73)*	1.64 (1.12, 2.41)*	
Subgroup						
Stratified by race/ethnicity						0.701
(Ref=no SRLM)						
Hispanics	265	1,752	3.02 (1.96, 4.66)*	2.02 (1.16, 3.52)*	1.39 (0.78, 2.45)	
Non-Hispanic Whites	360	3,081	2.42 (1.72, 3.42)*	2.08 (1.45, 2.98)*	1.82 (1.26, 2.63)*	
Non-Hispanic Blacks	321	1,485	2.28 (1.57, 3.32)*	1.85 (1.21, 2.82)*	1.57 (1.03, 2.39)*	
Other race	39	267	2.32 (0.99, 5.44)	1.04 (0.33, 3.33)	0.94 (0.27, 3.21)	
Stratified by gender						0.453
(Ref=no SRLM)						
Male	517	3,375	1.97 (1.22, 3.17)*	1.74 (0.98, 3.07)	1.46 (0.73, 2.90)	
Female	468	3,210	2.70 (2.01, 3.61)*	2.18 (1.58, 3.01)*	1.77 (1.31, 2.38)*	
Stratified by age						0.077
(Ref=no SRLM)						
18-65 years	550	5,345	3.06 (2.03, 4.60)*	3.03 (1.95, 4.71)*	2.22 (1.27, 3.87)*	
≥65 years	435	1,240	1.06 (0.77, 1.47)	1.07 (0.74, 1.54)	1.02 (0.62, 1.67)	

**Table 6:** Mediation analysis for the association between SRLM (defined as leg jerks or leg cramps) and risk of DM among race/ethnicity, gender, and age subgroups Analyses of the total population were adjusted for sociodemographics (age, gender, race/ethnicity, education, marital status, insurance, and family income to poverty ratio), body mass index, hypertension, and sleep apnea. Race, gender, and age were removed from the adjusted covariates of the corresponding stratified analyses. ^†^ Nonsignificant proportions of mediation were not shown. * p<0.05 SRLM: sleep-related leg movements; DM: diabetes mellitus; CRP: C-reactive protein; ADE: average direct effect; ACME: average causal mediation effect; CI: confidence interval

Mediator	DM		Total effect		ADE		ACME		Proportion mediated (%)^†^
	Yes	No	β	95% CI	β	95% CI	β	95% CI	
Short sleep duration									
Total population	985	6,585	0.0392	0.0163, 0.0639	0.0371	0.0146, 0.0609	0.0021	-0.0003, 0.0046	-
Hispanic	265	1,752	0.0528	0.0061, 0.1101	0.057	0.0082, 0.1147	-0.0043	-0.0110, 0.0001	-
Non-Hispanic Whites	360	3,081	0.0562	0.0255, 0.0890	0.0498	0.0212, 0.0820	0.0063	0.0021, 0.0113	11.39 (4.50, 21.73)*
Non-Hispanic Blacks	321	1,485	0.0762	0.0273, 0.1272	0.0775	0.0289, 0.1272	-0.0013	-0.0056, 0.0024	-
Other race	39	267	0.0174	-0.0599, 0.1036	0.022	-0.0582, 0.1098	-0.0045	-0.0269, 0.0119	-
Male	517	3,375	0.0449	-0.0053, 0.1011	0.0424	-0.0074, 0.0977	0.0025	-0.0022, 0.0078	-
Female	468	3,210	0.0639	0.0357, 0.0936	0.0599	0.0328, 0.0893	0.004	0.0012, 0.0078	6.17 (2.20, 12.44)*
18-65 years	550	5,345	0.0789	0.0399, 0.1214	0.0758	0.0368, 0.1173	0.0031	-0.0004, 0.0069	-
≥65 years	435	1,240	0.0103	-0.0457, 0.0690	0.0078	-0.0472, 0.0633	0.0024	-0.0030, 0.0090	-
Inflammation (CRP)									
Total population	928	6,138	0.0388	0.0158, 0.0612	0.0372	0.0143, 0.0601	0.0016	0.0003, 0.0036	4.14 (0.78, 13.25)*
Hispanic	254	1,651	0.0315	-0.0126, 0.0823	0.0309	-0.0131, 0.0800	0.0006	-0.0023, 0.0039	-
Non-Hispanic Whites	348	2,916	0.0426	0.0182, 0.0692	0.0408	0.0161, 0.0675	0.0019	0.0003, 0.0041	4.22 (0.50, 12.94)*
Non-Hispanic Blacks	290	1,321	0.0437	0.0095, 0.0848	0.0441	0.0104, 0.0845	-0.0004	-0.0044, 0.0033	-
Other race	36	250	-0.0017	-0.0676, 0.0884	-0.0006	-0.0647, 0.0902	-0.0011	-0.0076, 0.0031	-
Male	494	3,149	0.0267	-0.0192, 0.0813	0.0241	-0.0206, 0.0789	0.0026	0.0002, 0.0064	-
Female	434	2,989	0.0463	0.0270, 0.0674	0.0454	0.0263, 0.0662	0.0008	-0.0005, 0.0029	-
18-65 years	521	4,978	0.0569	0.0214, 0.0959	0.055	0.0203, 0.0941	0.002	0.00002, 0.0045	3.42 (0.06, 10.07)*
≥65 years	407	1,160	0.0042	-0.0541, 0.0649	0.0073	-0.0532, 0.0705	-0.0031	-0.0090, 0.0012	-

Introducing iron deficiency as an additional adjustment, only slightly attenuated the associations between SRLM and risk of DM in females aged 18-49 years, regardless of the SRLM definition used (Tables 7-8).

**Table 7:** Associations between SRLM (defined only with leg jerks) and risk of DM among females (additional adjustment for iron deficiency) Model 1: Adjusted for socio-demographics, including age, gender, race/ethnicity, education, marital status, insurance, and family income to poverty ratio. Model 2: Adjusted for socio-demographics and iron deficiency (iron deficiency included 2,287 missing values among females, which were taken as a separate group). Model 3: Adjusted for socio-demographics, body mass index, hypertension, sleep apnea, and iron deficiency. Race, gender, and age were removed from the adjusted covariates of the corresponding stratified analyses. ^†^The CI extends from zero to infinity due to too few sample sizes and too many covariates. * p<0.05 SRLM: sleep-related leg movements; DM: diabetes mellitus; OR: odds ratio; CI: confidence interval

Group	DM		Unadjusted model: OR (95% CI)	Model 1: OR (95% CI)	Model 2: OR (95% CI)	Model 3: OR (95% CI)
	Yes	No				
SRLM (ref=no)						
Female	627	3,901	2.09 (1.50, 2.92)*	2.23 (1.49, 3.32)*	2.19 (1.44, 3.31)*	1.79 (1.06, 3.03)*
Subgroup						
Stratified by race/ethnicity						
(Ref=no SRLM)						
Hispanic female	174	1,035	2.15 (1.37, 3.38)*	1.62 (0.93, 2.82)	1.50 (0.85, 2.66)	1.12 (0.50, 2.51)
Non-Hispanic White female	210	1,827	2.33 (1.56, 3.49)*	2.42 (1.53, 3.82)*	2.44 (1.51, 3.94)*	2.13 (1.32, 3.44)*
Non-Hispanic Black female	219	879	1.66 (0.87, 3.18)	1.47 (0.71, 3.05)	1.49 (0.71, 3.13)	1.71 (-)^†^
Other race females	45	139	3.25 (0.80, 13.29)	3.53 (0.57, 21.94)	2.83 (0.37, 21.79)	1.45 (-)^†^

**Table 8:** Associations between SRLM (defined as leg jerks or leg cramps) and risk of DM among females (additional adjustment for iron deficiency) Model 1: Adjusted for socio-demographics, including age, gender, race/ethnicity, education, marital status, insurance, and family income to poverty ratio. Model 2: Adjusted for socio-demographics and iron deficiency (iron deficiency included 2,287 missing values, which were taken as a separate group). Model 3: Adjusted for socio-demographics, body mass index, hypertension, sleep apnea, and iron deficiency. Race, gender, and age were removed from the adjusted covariates of the corresponding stratified analyses. ^†^The CI extends from zero to infinity due to too few sample sizes and too many covariates. * p<0.05 SRLM: sleep-related leg movements; DM: diabetes mellitus; OR: odds ratio; CI: confidence interval

Group	DM		Unadjusted model: OR (95% CI)	Model 1: OR (95% CI)	Model 2: OR (95% CI)	Model 3: OR (95% CI)
	Yes	No				
SRLM (ref=no)						
Female	468	3,210	2.70 (2.01, 3.61)*	2.18 (1.58, 3.01)*	2.13 (1.52, 2.98)*	1.75 (1.20, 2.53)*
Subgroup						
Stratified by Race/Ethnicity						
(Ref=no SRLM)						
Hispanic female	134	861	3.21 (2.03, 5.56)*	1.96 (1.07, 3.58)*	1.84 (1.02, 3.29)*	1.33 (0.56, 3.12)
Non-Hispanic White female	157	1,501	2.87 (2.00, 4.12)*	2.33 (1.55, 3.49)*	2.32 (1.52, 3.55)*	1.95 (1.40, 2.73)*
Non-Hispanic Black female	155	718	2.56 (1.59, 4.13)*	1.84 (1.09, 3.11)*	1.83 (1.07, 3.14)*	1.79 (-)^†^
Other race female	22	130	2.84 (0.79, 10.25)	1.28 (0.26, 6.34)	1.07 (0.17, 6.77)	0.91 (-)^†^

## Discussion

Our population-based study, based on a representative national cohort, identifies a significant association between SRLM and the prevalence of DM. After adjusting for known confounders such as age, BMI, sleep apnea, and hypertension, those reporting SRLM had a 72% increased likelihood of having DM compared to those without SRLM. Importantly, a dose-response relationship was observed, where a higher frequency of SRLM was linked to an increased prevalence of DM. Our exploratory mediation analyses suggested that short sleep duration and inflammation, as indicated by CRP levels, play roles in this association, albeit modestly, and these effects were notably more pronounced in non-Hispanic White individuals. These findings underscore the potential of SRLM as an indicator of DM risk and highlight the importance of considering demographic characteristics to fully understand these associations.

This study enhances our understanding of the association between SRLM and DM. We observed a strong association even after adjustment for key confounders (e.g., age, obesity, hypertension). This association displayed a dose-response pattern and is consistent across our large, diverse cohort that represents the general population, not just those typically at higher risk for RLS or PLMS. Historically, research on this relationship often requires complex clinical assessments or polysomnography and is focused on RLS or PLMS [7]. Our findings extend beyond these conditions by highlighting simple indicators such as “leg jerks” as potential markers for increased DM risk. If future longitudinal studies confirm our results, assessing “leg jerks” could be a valuable, straightforward tool for earlier detection and intervention in diabetes management. Given the simplicity of asking patients about frequent nocturnal leg jerks - a question that requires no specialized equipment or advanced training - this marker could be easily integrated into routine clinical evaluations. This approach may serve as an additional, low-cost screening tool to identify individuals who need closer monitoring of metabolic health. Such screening may be valuable in primary care or community health settings where access to sleep studies or laboratory testing may be limited.

The results of our study suggest the relationship between SRLM and DM was particularly evident among non-Hispanic White individuals, females, and younger adults. This differential impact prompts further exploration into the biological or environmental factors that contribute to these variations. For example, previous research has shown that PLMS and RLS are more common in non-Hispanic White individuals than in other racial/ethnic groups [19], which might partially explain the significant association observed in our study. Additionally, the variation in SRLM-DM relationships across different subgroups may be influenced by specific comorbidities that are prevalent within these groups. For instance, women with certain neurological conditions such as Parkinson’s disease and multiple sclerosis are known to have a higher incidence of RLS than men [7]. This interplay suggested a complex relationship between gender, neurological disease status, and sleep disturbances. Our findings also highlight an attenuated association of SRLM with DM in older adults, males, and non-White individuals, potentially due to smaller sample sizes of these subgroups or the criteria used to define prevalent DM. These subgroup differences underline the importance of tailored public health strategies that consider demographic and clinical characteristics when assessing the risk and management of DM. They also highlight the importance of using precise diagnostic criteria in future research to avoid potential confounding effects that could influence the interpretation of the relationship between SRLM and DM.

Our findings illustrate the mechanisms linking SRLM and DM, particularly through short sleep duration and inflammation. Short sleep duration impairs insulin sensitivity and disrupts hunger and satiety signals, potentially escalating insulin and glucose homeostasis and increasing inflammation [20]. Such physiological changes can promote unhealthy eating habits and increased calorie intake, known risk factors for DM. Individuals with SRLM often experience compounded sleep issues such as difficulty initiating and maintaining sleep, leading to sleep deprivation [21]. This state exacerbates sympathetic activity and oxidative stress, contributing to vascular endothelial dysfunction, a key player in diabetic complications [22]. Moreover, chronic inflammation, indicated by elevated CRP levels, is linked with metabolic disturbances [23,24], while observational studies suggest that conditions within the SRLM spectrum, like RLS, are associated with higher CRP levels; the precise relationship between SRLM and DM involves multiple pathways [25]. Although our study confirms modest mediation by sleep duration and inflammation, the data suggest the need to explore additional mechanisms. One such pathway could involve sympathetic nervous system overactivity, which may impair insulin sensitivity [26]. If SRLM is confirmed as a significant factor in DM risk, further research should investigate modifiable mechanisms to identify new intervention targets for reducing DM risk.

The influence of short sleep duration and inflammation on the link between SRLM and DM appears to be contingent upon racial and ethnic backgrounds. Notably, we observed that short sleep duration and CRP levels played a more significant mediating role in non-Hispanic White individuals compared to other race and ethnic groups. This variation may suggest that the pathway linking SRLM to DM is not uniform across all populations. Factors such as genetic predispositions, environmental influences, or access to healthcare might modify how these mediators affect the risk of DM [27-29]. Therefore, understanding these differing pathways is critical, especially when assessing SRLM in individuals at higher risk of DM. Further research is needed to explore these distinct mediation mechanisms and their implication for diabetes management and prevention strategies.

Our study has several strengths, including its reliance on a nationally representative sample and an analytical approach that adjusts for key confounders and assesses the dose-response relationship. However, certain limitations merit attention. First, our retrospective, cross-sectional design constrains our ability to establish causality. Despite this limitation, cross-sectional studies are invaluable for uncovering associations and formulating hypotheses that can be further tested in longitudinal studies. Second, we primarily self-reported data to assess SRLM and sleep duration, which may introduce biases. Although self-reports are prone to inaccuracies, they serve as essential tools in large-scale studies where more precise measures such as actigraphy are often unfeasible due to logistical and financial constraints. Future studies should validate self-reported data against objective clinical assessments, potentially using new devices like the RestEaze, which accurately measures leg movement during sleep [30]. Third, the data were collected between 2005 and 2008; however, the persistent nature of the associations between SRLM and DM across time suggests that these findings are still applicable. The extensive sample size of our study also facilitates a comprehensive examination of various confounders, potential mediators, and effect modifiers in the SRLM-DM relationship. Fourth, our analysis can’t differentiate between type 1 and type 2 DM, limiting our findings’ specificity to diabetes subtypes. Future research should stratify by diabetes type to better delineate the specific roles of SRLM in each. Furthermore, although we adjusted for multiple demographics, lifestyle, and clinical factors, the possibility of residual confounding from unmeasured or misclassified variables, such as unrecognized sleep disorders and medication use, cannot be ruled out and should be addressed in future studies. Lastly, assessments of both SRLM and sleep duration were made at a single time point, ignoring potential variations in the severity of SRLM or changes in sleep patterns over time. Future research should incorporate longitudinal measurements and use objective tools to track SRLM, enhancing the validity and reliability of these findings.

## Conclusions

In conclusion, our study adds to the growing evidence linking SRLM and an increased risk of DM. Our findings revealed that short sleep duration and inflammation play a significant mediating role in this association. Given the complexity of these relationships, prioritization of longitudinal studies is crucial for establishing causality and gaining deeper insights into the underlying mechanisms.

## References

[1] St-Onge MP, Grandner MA, Brown D, Conroy MB, Jean-Louis G, Coons M, Bhatt DL (2016). Sleep duration and quality: impact on lifestyle behaviors and cardiometabolic health: a scientific statement from the American Heart Association. Circulation.

[2] Lloyd-Jones DM, Allen NB, Anderson CA (2022). Life’s Essential 8: updating and enhancing the American Heart Association’s construct of cardiovascular health: a presidential advisory from the American Heart Association. Circulation.

[3] Phillips B, Young T, Finn L, Asher K, Hening WA, Purvis C (2000). Epidemiology of restless legs symptoms in adults. Arch Intern Med.

[4] Ekbom K, Ulfberg J (2009). Restless legs syndrome. J Intern Med.

[5] Zobeiri M, Shokoohi A (2014). Restless leg syndrome in diabetics compared with normal controls. Sleep Disord.

[6] Merlino G, Fratticci L, Valente M (2007). Association of restless legs syndrome in type 2 diabetes: a case-control study. Sleep.

[7] Ning P, Mu X, Yang X, Li T, Xu Y (2022). Prevalence of restless legs syndrome in people with diabetes mellitus: a pooling analysis of observational studies. EClinicalMedicine.

[8] Ding Q, Qin L, Wojeck B (2021). Polysomnographic phenotypes of obstructive sleep apnea and incident type 2 diabetes: results from the DREAM study. Ann Am Thorac Soc.

[9] Grandner MA, Winkelman JW (2017). Nocturnal leg cramps: prevalence and associations with demographics, sleep disturbance symptoms, medical conditions, and cardiometabolic risk factors. PLoS One.

[10] Moore JX, Chaudhary N, Akinyemiju T (2017). Metabolic syndrome prevalence by race/ethnicity and sex in the United States, National Health and Nutrition Examination Survey, 1988-2012. Prev Chronic Dis.

[11] Trotti LM, Rye DB, De Staercke C, Hooper WC, Quyyumi A, Bliwise DL (2012). Elevated C-reactive protein is associated with severe periodic leg movements of sleep in patients with restless legs syndrome. Brain Behav Immun.

[12] Tsalamandris S, Antonopoulos AS, Oikonomou E (2019). The role of inflammation in diabetes: current concepts and future perspectives. Eur Cardiol.

[13] (2023). National Health and Nutrition Examination Survey data. Hyattsville, MD: U.S. Department of Health.

[14] American Diabetes Association Professional Practice Committee (2022). 2. Classification and diagnosis of diabetes: Standards of Medical Care in Diabetes - 2022. Diabetes Care.

[15] Zhang H, Zhang Y, Ren R (2022). Polysomnographic features of idiopathic restless legs syndrome: a systematic review and meta-analysis of 13 sleep parameters and 23 leg movement parameters. J Clin Sleep Med.

[16] Shan Z, Ma H, Xie M (2015). Sleep duration and risk of type 2 diabetes: a meta-analysis of prospective studies. Diabetes Care.

[17] Krittanawong C, Kumar A, Wang Z (2020). Sleep duration and cardiovascular health in a representative community population (from NHANES, 2005 to 2016). Am J Cardiol.

[18] Trotti LM, Becker LA (2019). Iron for the treatment of restless legs syndrome. Cochrane Database Syst Rev.

[19] Doan TT, Koo BB, Ogilvie RP, Redline S, Lutsey PL (2018). Restless legs syndrome and periodic limb movements during sleep in the Multi-Ethnic Study of Atherosclerosis. Sleep.

[20] Rogers EM, Banks NF, Jenkins ND (2024). The effects of sleep disruption on metabolism, hunger, and satiety, and the influence of psychosocial stress and exercise: a narrative review. Diabetes Metab Res Rev.

[21] Karna B, Sankari A, Tatikonda G (2025). Sleep Disorder. StatPearls [Internet].

[22] Eisensehr I, Ehrenberg BL, Noachtar S (2003). Different sleep characteristics in restless legs syndrome and periodic limb movement disorder. Sleep Med.

[23] Singh B, Goyal A, Patel BC (2025). C-reactive Protein: Clinical Relevance and Interpretation. StatPearls [Internet].

[24] Ridker PM, Rifai N, Rose L, Buring JE, Cook NR (2002). Comparison of C-reactive protein and low-density lipoprotein cholesterol levels in the prediction of first cardiovascular events. N Engl J Med.

[25] Joseph V, Nagalli S (2025). Periodic Limb Movement Disorder. StatPearls [Internet].

[26] Olgun Yazar H, Yazar T, Özdemir S, Kasko Arici Y (2019). Serum C-reactive protein/albumin ratio and restless legs syndrome. Sleep Med.

[27] MacKinnon DP, Krull JL, Lockwood CM (2000). Equivalence of the mediation, confounding and suppression effect. Prev Sci.

[28] Hill-Briggs F, Adler NE, Berkowitz SA (2020). Social determinants of health and diabetes: a scientific review. Diabetes Care.

[29] Darraj A (2023). The link between sleeping and type 2 diabetes: a systematic review. Cureus.

[30] Anyfantakis D, Katsanikaki F, Symvoulakis EK (2020). Diabetic neuropathy and restless legs syndrome: can a known chronic condition slow down our diagnostic way of thinking? A case report and a short literature overview. Med Pharm Rep.

